# *Cerebral venous sinus thrombosis following* ChAdOx1 nCoV-19 AstraZeneca COVID-19 vaccine

**DOI:** 10.1177/20480040231169464

**Published:** 2023-04-11

**Authors:** Shyam S Sharma, Giosue Gulli, Pankaj Sharma

**Affiliations:** 1 Edinburgh Medical School, University of Edinburgh, Edinburgh, UK; 2 Department of Stroke Medicine, Ashford & St Peter's Hospitals NHS Foundation Trust, Surry, UK; 3 Institute of Cardiovascular Research, Royal Holloway University of London (ICR2UL), London, UK; 4 Department of Clinical Neuroscience, Imperial College Healthcare NHS Trust, London, UK

**Keywords:** Acute stroke syndromes, COVID-19, vaccination, cerebral venous thrombosis

## Abstract

A woman in her mid-twenties was admitted with headache, ultimately leading to a diagnosis of cerebral venous sinus thrombosis 10 days after receiving a first dose of the AstraZeneca ChAdOx1 nCoV-19 vaccine (Vaxzevria). We report this case from clinical investigations to outcomes and discuss the issues raised by it regarding the ChAdOx1 nCoV-19 vaccine.

## Background

The COVID-19 pandemic has resulted in considerable excess of mortality across the world and since vaccines have become approved for use, there have been multiple reports of severe adverse effects.

The ChAdOx1 nCoV-19 Oxford/Astra Zeneca vaccine is a replication-deficient chimpanzee adenoviral vector vaccine which expresses the SARS-CoV-2 spike protein gene.^1^ Clinical trials showed an efficacy of 64.1% after one standard dose and 70.4% after two doses, varying between interval times and doses. There were 175 severe adverse events reported (84 events in the ChAdOx1 nCoV-19 group, 91 in the control group), of which 3 were classified as possibly being related to the vaccine (1 in the ChAdOx1 nCoV-19 group, 1 in the control group, 1 remained unmasked).^2^

By the end of June 2022, 443 cases of thrombosis with thrombocytopenia were reported in an estimated 49 million doses.^3^ The Joint Committee on Vaccination and Immunisation (JCVI) advised that under-30's in the UK are to be offered an alternative vaccination to the ChAdOx1 nCoV-19 vaccine due to the evidence linking it to rare blood clots, after analysing the available data on epidemiology, benefit-risk profile by age, modelling predictions on future disease trends and the current forecast on vaccine supply.^4^

We report a case of cerebral venous sinus thrombosis (CVST) following the use of the AstraZeneca COVID-19 vaccine and discuss how a diagnosis may be made and its management.

## Case presentation

A woman in her mid-twenties received her first dose of ChAdOx1 nCoV-19 vaccination at the beginning of 2021. Ten days following the vaccination, she presented to the Emergency Department having been unwell for around one week, with a severe headache developing over the previous few days accompanied by nausea and vomiting.

On the day of admission, she suffered a generalised tonic-clonic seizure. After regaining consciousness, she suffered slurring of speech and left-hand clumsiness. She complained of sensory discomfort affecting the left arm and some dysarthria, and reported moderate to severe headache with mild neck stiffness. The patient had a history of mild asthma, otherwise reported no relevant medical or surgical history. Apart from a once-daily contraceptive pill and salbutamol puffs when required, she was not taking any medications regularly.

On examination, her Glasgow Coma Score was 15, blood pressure was 153/106 mmHg with a pulse of 130 beats per minute, temperature was 36.8 °C, respiratory rate 18 breaths per minute, and SpO_2_ was 97%. Limb power and reflexes were normal. Other examinations of cardiac, respiratory, and abdominal organs were unremarkable.

About seven hours following admission, the patient experienced another episode of loss of consciousness with seizure markers. She was given levetiracetam 1 g intravenously. Clinical examination after regaining consciousness found no new focal neurological signs.

Her blood tests on admission revealed mildly elevated C-reactive protein (35 mg/L), raised white cell count (WCC – 15.1 × 10^9^/L) and neutrophils (13.2 × 10^9^/L). Pregnancy test and COVID-PCR tests were negative. Previously undetected subclinical hyperthyroidism was noted with thyroid stimulating hormone at 0.01 mIU/L and free thyroxine (FT4) at 29.5 pmol/L. Following a specialist endocrine assessment this was felt to be incidental, and she was commenced on 15 mg Carbimazole once daily.^5^ Liver function test, kidney function, bone profile, rheumatoid factor, clotting function and coagulation screen were otherwise unremarkable. The patient's platelet count was normal (223 K/µL) and remained such during her in-patient stay, ranging from 185 to 228 K/µL.

Brain MRI supported a diagnosis of CVST for which she was commenced on therapeutic doses (1 mg/kg) of low-molecular weight heparin (LMWH) every 12 hours.

**Figure 1.**
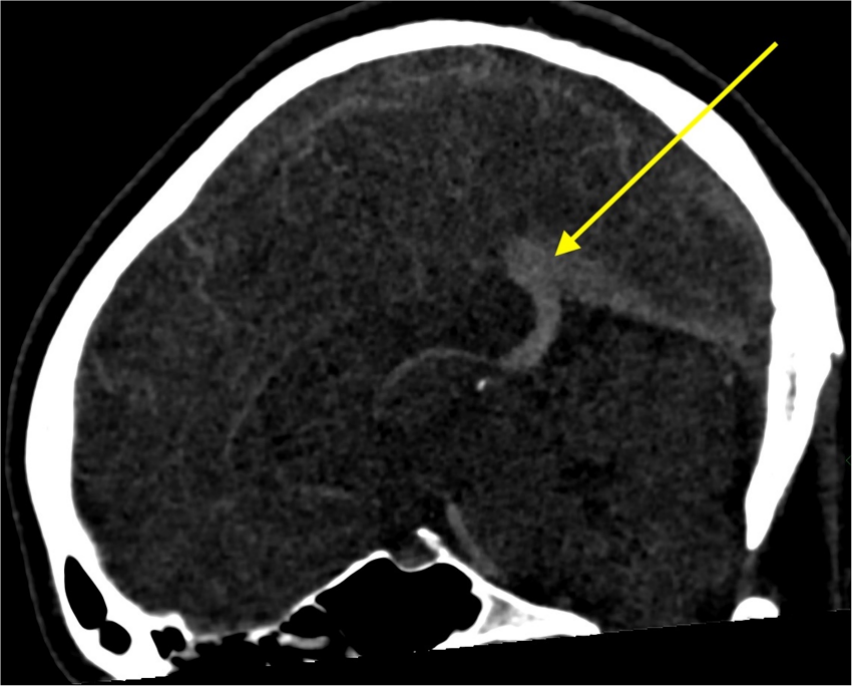
CT venography showing venous thrombosis.

A computed tomography venogram confirmed the presence of extensive CVST involving the sagittal sinus, the left transverse sinus and to a lesser extent the right transverse sinus (Figure 1). No intraparenchymal abnormalities were observed. An MRI scan of her brain with venogram (MRV) sequences confirmed the presence of extensive CVST without any parenchymal involvement.

In the following days, the patient's symptoms of nausea and headache progressively improved. A full CT body scan excluded any presence of malignancy.

## Outcome and follow-up

She was discharged after four days with a mild headache but feeling subjectively better. At discharge, there was a plan to switch the anticoagulation from LMWH to oral vitamin K antagonist anticoagulation (warfarin), varying the dose to keep the International Normalised Ratio between 2 and 3 units, and review her in 6 months with a repeat MRV. It was recommended to suspend use of the contraceptive pill.

After 5 weeks, the patient contacted the stroke department asking whether it was safe for her to have the second dose of the COVID-19 vaccination. At this point the patient disclosed that she received the first dose of the AstraZeneca vaccine ten days prior to admission. It was deemed not appropriate to receive the second dose of the ChAdOx1 nCoV-19 vaccination, and an alternative vaccine was recommended. Later, national guidelines were released dictating that the second dose of the ChAdOx1 nCoV-19 vaccination should still be given in the absence of thrombocytopenia, unless the patient is under 30 without any underlying health problems.^4^

## Discussion

We present the case of a woman in her mid-twenties with confirmed CVST within ten days of receiving a first dose of a ChAdOx1 nCoV-19 vaccination.

CVST has a wide spectrum of clinical presentation, ranging from headache with papilledema to focal deficit, seizures, and coma. CVSTs had a reported incidence rate of 2.6 per million and mortality rate of ∼4.5% before COVID-19.^6,7^ Recently, it, alongside thrombocytopenia, has been linked to the ChAdOx1 nCoV-19 vaccine. In CVST cases with thrombocytopenia after vaccination, the mortality rate decreased from 47%, in those occurring prior to 28 March 2021, to 22% in cases after this date.^8^ The mortality rate in cases of thrombosis without thrombocytopenia following a vaccination was reported to be 16%.^9^

### Pathophysiology

Other papers have reported a similarity between the presentation of patients with vaccine-induced thrombotic thrombocytopenia (VITT) and those with heparin-induced thrombocytopenia (HIT); however, these patients had often no known exposure to heparin before hospitalisation.^1,10,11^ HIT is caused by the production of IgG class, platelet-activating antibodies that recognise heparin and platelet factor 4 (PF4) complexes (PF4/H) or multimolecular complexes of PF4 bound to other polyanions.^12,13^ HIT patients often, but not always, test positive for the antibodies against PF4 (using an Enzyme-Linked Immunosorbent Assay), which is also common among patients who presented with clotting and low platelet count 5 to 16 days after vaccination, suggesting that the mechanism of clotting caused by the ChAdOx1 nCoV-19 vaccine is similar to that of HIT.^14^ The free DNA in the vaccine may be a potential trigger, as it is an example of a polyanion that has been shown to be able to induce conformational changes in PF4 which exposes the HIT antigens and produce clinical consequences similar to those of HIT.^13^

This thrombotic thrombocytopenia could also be caused simply by an autoimmune reaction induced by SARS-CoV-2. Several studies have reported that anti-PF4/H antibodies are present in COVID-19 patients which may be the cause of patients, both with severe COVID-19 and after vaccination, presenting similarly to autoimmune HIT.^15^

Another potential mechanism is platelet activation by adenovirus. Due to adenovirus being a non-blood borne pathogen, it is unable to survive in the body and therefore binds to blood cells. Platelets are the predominant adenovirus binding blood cell type which then become activated. This activates the coagulation cascade, causing thrombocytopenia and disseminated intravascular coagulation.^16^ However, a large number of cases presenting with thrombotic thrombocytopenia following the ChAdOx1 nCoV-19 vaccine, test positive for anti-PF4 antibodies which suggests that this alternative mechanism is unlikely due to adenovirus not interacting with PF4. Furthermore, this theory relies on significant amounts of vaccine particles reaching the bloodstream after intramuscular injection, which is unlikely. However, the rate of CVST after the second dose of the ChAdOx1 vaccine is estimated to be 1.8 per million or smaller, implying that it is not a result of the immune response to the spike protein.^17,18^

Recently the first hypothesis has had more support including from Greinacher et al. who demonstrated that vaccine components formed antigenic complexes with PF4 on the platelet surfaces, which were bound by anti-PF4 antibodies. They also found that DNA increased the size of the PF4 complexes that was formed with the vaccine components and the antibodies. The size decreased with the addition of heparin and the negative charge of the complex was neutralised not by this addition but by the addition of PF4, all of which indicates that the complex formation is charge driven.^19^

### CVST cases post-AstraZeneca vaccine

Other researchers have recorded three female patients of childbearing age in Germany who presented with intracranial venous sinus thrombosis after receiving the first dose of the ChAdOx1 nCoV-19 vaccine. All three women denied using oral contraception and all presented with thrombocytopenia (between 60 and 92 K/µL). The three patients were treated with LMWH. The clinical presentation for these cases was similar to each other and to the case we report; the patients presented with severe headaches 4–8 days after receiving the vaccine following which their condition worsened acutely. One 22-year-old woman was reported to have suffered from a self-limited generalised epileptic seizure, near identical to our patient.^20^

Another case series followed 11 patients, one of which presented with fatal cerebral haemorrhage and died. Of the 10 patients for which there is data, only one presented with a platelet count over 100 K/µL, which is still classified as thrombocytopenia (<150 K/µL).^10,21^

### Non-VITT (thrombosis without thrombocytopenia following a vaccine) CVST cases post-vaccination

Although there is increasing understanding to suggest VITT has more severe effects and a higher mortality rate, it is still important to understand the reasoning behind thrombosis post-vaccination without the presence of thrombocytopenia, as the difference in pathophysiology could potentially lead to a need for a different management approach.

Unfortunately, due to a combination of factors such as the patient not presenting with thrombocytopenia, not disclosing that she received the first dose of vaccination until after her discharge and being so early in the COVID pandemic, there were no guidelines or precedence, and no further tests were conducted that may have helped to shed light on non-VITT. Currently, the exact pathophysiology of non-VITT is unknown but there are a few potential mechanisms.

One suggested method of thrombosis in the absence of thrombocytopenia potentially relates to adenovirus-based vaccines causing a local inflammatory response and platelet aggregation. When combined with local predisposing factors such as blood stasis, may lead to venous thrombosis without thrombocytopenia.^22^ However, should patients with non-VITT show to have high levels of anti-PF4 antibodies, this hypothesis does lose some merit, as this mechanism does not interact with PF4.

Another hypothesised method is that non-VITT is caused by the same mechanism as VITT but low concentrations of PF4 cause local platelet aggregation where there are already the aforementioned predisposing factors.^22^

Indeed, many thrombosis cases without thrombocytopenia after vaccination do not seem to have elevated anti-PF4 antibody levels.^22,23^

If the mechanism of the condition is different to the well-documented VITT, the question of its treatment and management arises. Below, we discuss current guidelines for VITT, non-VITT and how these have changed since March 2021.

### Current guidelines

Current UK Government guidelines advise that any patient presenting with thrombosis and thrombocytopenia following the ChAdOx1 nCoV-19 vaccine should have their second dose delayed until their clotting has stabilised, and they should then complete the primary course of vaccination.^17^ These guidelines came into force after our patient was recommended to not receive the second dose of the ChAdOx1 nCoV-19 vaccine.

NICE guidelines acknowledge the lack of evidence in the management of VITT, but reached a consensus for the use of intravenous immunoglobulin in the primary treatment of VITT patients.^24^

A study by The Oxford Vaccine Group's Com-Cov has shown that those who received a dose of Pfizer/BioNTech BNT162b2 28 days after a dose of ChAdOx1 nCoV-19 had an immune response comparable to having two doses of BNT162b2. Their mean concentrations of SARS-CoV-2 anti-spike IgG antibodies was 9.2 times greater than those who received the two standard ChAdOx1 nCoV-19 doses.^25^ This suggests that recommending a second dose of a different vaccine would provide a stronger response to COVID without putting the patient at higher risks of adverse effects.

At the time of our case, there were no guidelines to treatment and management of VITT patients. In April 2021, guidelines were published that stated the second dose of the AstraZeneca vaccine should be offered to all those who received the first dose. These guidelines were still included in the updated guidance published in February 2022.^26^

National guidelines have hardly changed since they were first released in April 2021 despite further research in this field. These guidelines do not explicitly recognise the phenomena of CVST without thrombocytopenia and therefore do not have any specific recommendations as to the management of this condition. Therefore, it is reasonable to assume that the management of this condition should follow that of CVST, with the addition of a thrombophilia screen to rule out VITT.

In March 2023, the UK Health Security Agency updated the ‘COVID-19: green book’ to state that there have been no confirmed cases reported in pregnant women. Due to this, and the seemingly autoimmune nature of VITT, women who are pregnant, in post-partum or on the contraceptive-pill seem to be at no higher risk of VITT from the AstraZeneca vaccine.^27^ As such, there is no specific guideline to offer these women a different vaccination. However, it does caution its use in those who have had a previous episode of HIT.^27^

A further area of interest is the efficacy of the management for this condition which is found in the NICE guidelines. These guidelines recommend a first line treatment of intravenous immunoglobulin and potentially performing a plasma exchange and adding steroids for those at higher risk of a poor prognosis.^24^ Although there is research showing that immunomodulation is linked to a lower mortality, it is unclear as to the efficacy of platelet transfusion and use of non-heparin anticoagulants, with some research showing lower mortality and others showing no significant difference.^9,28^

### Limitations

Several limitations need to be noted. Our patient was taking the oral contraceptive pill, a noted risk factor for CVST, with one study showing an odds risk ratio of 7.6 for oral contraceptive use.^29^ However, the temporal relationship of the venous thrombosis (within 10 days) and vaccination, along with many other reports of similar cases, makes it unlikely, although not impossible, to assign the aetiological cause of the CVST to the contraceptive pill.

Although most cases have reported CVST with thrombocytopenia, some cases have been reported in the absence of thrombocytopenia.^22,30^ In our case, the patient had a platelet count of 223 10^9^/L on admission. No PF4 levels were recorded as this relationship was not known at the time of presentation. However, not all cases with vaccine associated CVST have shown abnormal PF4 levels and the timeframe (10 days) of the event and the first vaccine dose meets current accepted criteria for aetiological cause attributable to ChAdOx1 nCoV-19 vaccination.

### Learning points

- The ChAdOx1 nCoV-19 vaccine has been shown to increase the risk of CVST.
- Headache within 28 days of the first dose of COVID-19 vaccine may warrant further investigation.
- The use of a PF4-dependent ELISA may be used to confirm the diagnosis of vaccine-induced thrombocytopenia, predicting the occurrence of a CVST, although clinicians should be wary that anti-PF4 antibodies may be absent in some non-VITT cases.
- Recommendation of receiving a subsequent dose of ChAdOx1 nCoV-19 vaccine should be carefully considered in those with thrombosis within the first 28 days even in the absence of thrombocytopenia.
